# The Successful Treatment of Cleft Palate

**Published:** 1895-08

**Authors:** Eugene F. Hoyt


ARTICLE II.

### THE SUCCESSFUL TREATMENT OF CLEFT PALATE.

BY EUGENE F. HOYT, M. D.

To become truly proficient in any specialty requires a protracted and complex experience, to the exclusion of all other irrelevant subjects. No man becomes a master at one bound, and while this is true of all activities, it is pre-eminently true of all measures which have for their object the relief of human suffering. Other things being equal, the longer experience brings the greater skill. The author of a beneficent operation is guilty of a crime against humanity if he fails to make his discovery known, and furthermore, unless such an operation is performed so frequently as to attract public attention,or unless public attention is frequently called to it by means which are regarded as unprofessional, hundreds, and possibly thousands, may go through life in ignorance that complete relief is within reach.

Cleft palate is a malady not alone disturbing to the unfortunate individual who bears it, but disturbing to all who come within the sound of his voice.

It is not his voice *per se* which is so disagreeable, but that voice, when it is used in speech, becomes in many instances a medley of inarticulate sounds.

The mental suffering of a sensitive person having such a deformity is in most cases more acute than that arising from almost any other congenital defect, and this is especially the case with a comely young woman who has no hare-lip and no external evidence of her deformity. Yet her speech betrays her and her mortification becomes unendurable.

Naturally the afflicted one turns to her medical adviser, and just as naturally he refers her to the surgeon; and for seventy-five years surgeons have been attempting to remedy the defect.

There is no excuse for any interference which does not correct the speech. No other function is disturbed sufficiently to justify remedial action.

It is quite safe to say that the surgical operation has been attempted not less than ten thousand times, and it is quite true that more than nine thousand five hundred of these cases have been complete failures in restoring normal speech.

It is quite possible for a skillful surgeon in particularly favorable cases to close the fissure by suture, and obtain a surgical success, but the operation is nevertheless a failure, because the function of articulate speech is not restored by a mere union of the divided palate.

Normal articulation cannot exist where there is inability to make a perfect contact of the palate with the pharynx, and thus close the passage from the larynx to the nose. In cleft palate there is an absence of tissue which cannot be created by surgery, and however perfectly the sides of the cleft may be brought together and united, the palate is always too short to close the pharyngeal passage, and perfect normal speech can never be acquired where that passage remains open. Therefore it is that even surgical successes almost invariably become functional failures.

These insurmountable obstacles have been frequently recognized and acknowledged by eminent surgeons, and because of the utter hopelessness of success, operations have been largely discouraged. Nevertheless, others more daring and reckless have tried to overcome the admitted difficulties by resorting to what, in the writer's estimation, are most barbarous methods.

One of the latest to claim attention takes the child unfortunate enough to be born with a cleft palate, within a few weeks after birth, and cuts away the edges of the fissure, bone and all; then passes wire through the jaw-bones, reaching across from side to side above the gap. These wires are then twisted until the bones give way and the gap is closed. If the resistance is too great, the bone is divided horizontally on either side with a heavy scalpel. Oh, Surgery, what crimes are not committed in thy name!

It is exactly thirty years since Dr. Norman W. Kingsley, at that time in London, but now at 115 Madison avenue, New York city, invented an artificial palate which was original in its conception, construction and application. This instrument not only replaced the missing portion of the palate, but, being flexible and under the muscular control of the adjacent parts, the function of normal articulation was also restored. Thus a defective natural organ of speech was made perfectly serviceable by an artificial one.

It has long since passed the period of experiment. It is a complete scientific and practical success. With it very many people who were deprived of entry into society and into business have been released from their bondage, and permitted to associate with their fellows upon complete equality.

During these thirty years Dr. Kingsley has applied this instrument in hundreds of cases, to persons of all ages, from the child of six years to the adult of sixty, and to every conceivable form of fissure.

With the means thus furnished by a properly adjusted artificial palate there is no more reason to anticipate failure than there is reason to believe that a person cannot learn to speak a language.

With the older cases there are usually bad habits of speech, which must be broken up and a new habit formed. These bad habits of speech are due to inability, but, with the defective organ restored, the difficulties cease to be insurmountable, as is proven by the large number who are now wearing such appliances, and who are not suspected of having a deformity by those who hear them speak. This is pre eminently a fact with children, and especially if the child can be placed under proper training for articulation.

It matters little whether the fissure is large or small; whether it be confined to the soft palate, or becomes more complicated and involves the roof of the mouth and a division of the maxillary bones; or whether or not it is associated with hare-lip; in every case the effect of the cleft is the same in character, differing only in degree, and the same principles apply to the correction, variations of the instrument being made to suit each individual case.—*The Brooklyn Medical Journal.*

